# Mechanisms causing size differences of the land hermit crab *Coenobita rugosus* among eco-islands in Southern Taiwan

**DOI:** 10.1371/journal.pone.0174319

**Published:** 2017-04-07

**Authors:** Chia-Hsuan Hsu, Keryea Soong

**Affiliations:** Department of Oceanography, National Sun Yat-sen University, Kaohsiung, Taiwan; University of California, UNITED STATES

## Abstract

Numerous environmental factors can influence body size. Comparing populations in different ecological contexts is one potential approach to elucidating the most critical of such factors. In the current study, we found that the body size of the land hermit crab *Coenobita rugosus* was significantly larger on Dongsha Island in the South China Sea than on other eco-islands around Southern Taiwan. We hypothesized that this could be due to differences in (1) shell resources, (2) parasite impact, (3) competition, (4) predation, and (5) food. We found no supporting evidence for the first three hypotheses; the shells used by the hermit crabs on Dongsha were in poorer condition than were those used elsewhere, extremely few individuals in the region had ectoparasites, and the density of hermit crabs varied considerably among localities within each island. However, significantly higher percentages of *C*. *rugosus* reached age 3 years on Dongsha than at Siziwan bay in Taiwan. Two growth rate indices inferred from size structures suggested faster growth on Dongsha than at Siziwan. The condition index (i.e., the body mass/shield length ratio of *C*. *rugosus*) was also greater on Dongsha than at Siziwan. Therefore, Dongsha hermit crabs seem to have superior diet and growth performance. Seagrass debris accumulation at the shore of Dongsha was considerable, whereas none was observed at Siziwan or on the other islands, where dicot leaves were the dominant food item for the vegetarian hermit crabs. We then experimentally evaluated the possible role of seagrass as food for *C*. *rugosus*. The crabs on Dongsha preferred seagrass to dicot leaves, and their growth increment was faster when they fed on seagrass than when they fed on dicot leaves; no such differences were found in the Siziwan hermit crabs. The aforementioned results are compatible with the food hypothesis explaining the size differences among the islands. The predator hypothesis could explain the greater life span but not the other findings. Populations of *C*. *rugosus* on islands with seagrass debris piles probably contribute more to the gene pool of the species because higher proportions of these populations could achieve high fecundity. The fate of these terrestrial hermit crabs may rely on the health of underwater seagrass ecosystems that are under threat from global change.

## Introduction

The body size of organisms has wide-ranging implications such as in the physiology, ecology, and evolution of species [1, 2]. Numerous factors could influence body size differences among different populations of a species [3]. The most recognized examples may be found in fisheries biology because overfishing can change population structures, shorten the life span of individuals, and impose selective pressure on life history traits, among other effects [4]. This is essentially because fish and invertebrate targets are more likely to be caught before reaching their full body size in overfished regions [5]. Such comparisons could be made either temporally (i.e., before vs. after fishing) or spatially (i.e., among regions with different fishing pressure) [6]. Many studies have examined both the ecological and evolutionary effects of reduced body size and life span due to overexploitation [7].

Food availability is also a potential contributing factor to body size differences among the populations of a species [8]. Rosenzweig’s hypothesis, for example, explains geographic variation in body sizes as a result of productivity pattern differences [9]. This could potentially apply to carnivores, herbivores, and omnivores [10–12].

Competition can significantly affect the body size of a population. This effect is both exhibited in nature and in the laboratory. Densities within populations may be a critical indicator of intraspecific competition [13]. Interspecific competition can also occur when sympatric species occupy similar habitats; their body sizes may deviate from those of allopatric populations in different sites [14, 15]. In such instances of character displacement, the absence or presence of other species plays a crucial role in affecting the body sizes of local populations.

Parasites may affect the body sizes of their hosts and in turn affect the body sizes of the host populations if the infection rates are sufficiently high [16]. Natural populations under stress from competition or lack of food are especially likely to exhibit the negative effects of parasitism, as demonstrated in a study of three-spined sticklebacks [17]. In New Zealand, the occurrence of sexual reproduction in a freshwater snail was shown to be dependent on the prevalence of parasites that vary substantially among rivers [18]. The growth rate difference between infected and noninfected individuals is often used to test the nature of the symbiosis between the host and the symbiotic species because parasites tend to absorb nutrients from hosts and inhibit their growth [16, 19].

Gastropod shell availability is a requirement particular to hermit crabs. Shells provide protection for hermit crabs, which are more likely to grow faster when adequate shells are readily available [20–22]. By contrast, a limited supply of shells can limit their growth and size [23–25]. The availability of gastropod shells differing among islands could explain body size differences among hermit crab populations [26].

In this study, we compared the body sizes of the land hermit crab *Coenobita rugosus* in various islands around Southern Taiwan. We then tested the various hypotheses explaining the body size differences among these populations.

## Materials & methods

### Size composition

**Fig 1:**
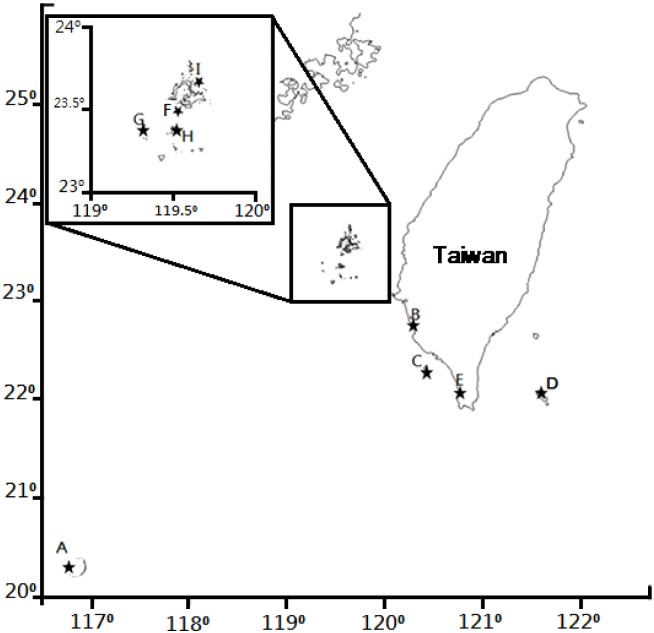
Islands/eco-islands of this study. A: Dongsha, B: Siziwan, C: Xiaoliuqiu, D: Lanyu, E: Howan, F: Tongpanyu, G: Huayu, H: Giang-jun-ou-yu, and I: Yuanbeiyu.

Seven islands, namely Dongsha (20°24'N, 116°43'E), Xiaoliuqiu (22°20'N,120°22'E), Lanyu (22°02'N, 121°32'E), Tongpanyu (23°30'N, 119°31'E), Huayu (23°24'N, 119°19'E), Giang-jun-ou-yu (23°22'N, 119°31'E), and Yuanbeiyu (23°38'N, 119°38'W), and two eco-islands, namely Siziwan (22°38'N, 120°15'E) and Howan (22°02'N, 120°41'E, Fig 1), were investigated between November 2013 and November 2014. The eco-islands are actually bays on Taiwan’s main island that are isolated from the others by uninhabitable habitats. The hermit crabs do not disperse to other eco-islands except by planktonic larvae through the sea. True islands and eco-islands are all referred to as islands in this study.

Baited traps were used to attract hermit crabs, including *C*. *rugosus*, the target of this study, at various locations. Rice bran was prepared using dry heating and stirred for approximately 5 min until fragrance was released. At each location, 100 mL of rice bran was scattered on selected spots 1 h before sunset. Two hours later, the attracted hermit crabs were collected in buckets. The shield length, used as a body size parameter, and the palm length of the left arm were recorded for each individual [27]. In addition, the shell conditions were recorded before the crabs were released to their original sites. When the shield length could not be obtained, it was estimated using the following regression formula: shield length = 0.74 × (palm length) + 0.09 cm [28].

We used the 95th percentile of population size as an index for comparing populations in different islands, with 95% confidence intervals (CIs) calculated in XLSTAT by using the resampling method with 10,000 repeat samples (https://www.xlstat.com/en/).

### Shell status

**Fig 2:**
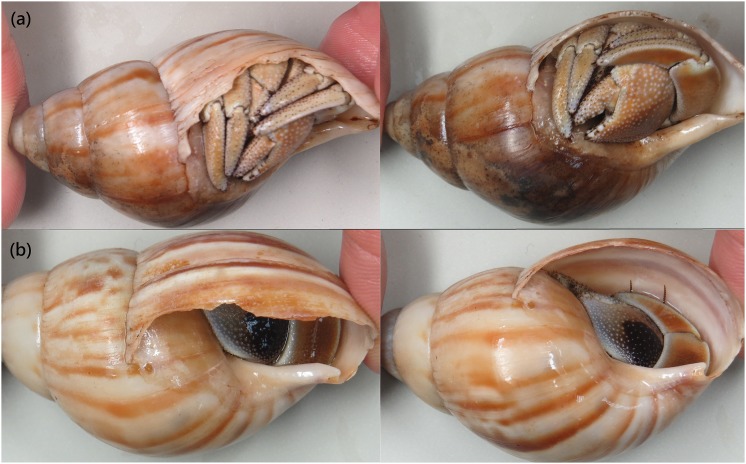
*Coenobita rugosus*. Identification of shell condition, with examples of (a) disadvantageous, upper row, and (b) advantageous shells, lower row.

Because hermit crabs with large and intact shells have higher growth rates [21, 29, 30] and hermit crabs tend to choose intact and suitable shells [31], a simple method was used to categorize individual shells as advantageous or disadvantageous. In advantageous shells, the left arms of the hermit crabs were within the plane of the aperture of the shells, whereas in disadvantageous shells, parts of the left arms protruded beyond the shell opening plane (Fig 2). The sites were compared using Chi-square tests.

### Cohort analysis

To compare the growth of hermit crabs, samples from Dongsha and Siziwan were collected using the same methods in November of both 2013 and 2014.

FISAT II software developed by ICLARM was used to analyze size structures of the populations for an objective assessment of the cohorts and their distributions [32]. The means and standard deviations of each cohort were estimated using a graphic separating normally distributed groups from a mixture of different cohorts [33]. The actual calculation was done by FISAT II.

Increments in shield length were used to compare the growth rates of the cohorts. Two approaches were adopted. The first was a horizontal approach, in which differences in means between cohorts 1 and 2 of the same years were used. The second was a vertical approach, in which the differences in means between cohort 2 of 2014 and cohort 1 of 2013 were used.

### Condition indices

The condition index (i.e., body mass / shield length) was calculated for each hermit crab, and then nonparametric statistics were used to test within a small size range to determine whether the Dongsha or Siziwan population had a higher index score. In addition, ANCOVA was used to compare the regression formula between the body weights and shield lengths of hermit crabs within the whole size ranges of the two sites. The hermit crabs for these analyses were collected within a week (i.e., June 30–July 2, 2014 for Dongsha and July 4–6, 2014 for Siziwan) to reduce any possible seasonal variation. After each crab was cleaned in water and blot-dried with tissue paper, it was weighed without its shell by using electronic scales.

### Food preference test

Two food items (i.e., seagrass debris from a Dongsha beach and dicot leaves from the Siziwan beach) were used. They were provided to the tested hermit crabs from either Dongsha or Siziwan. The food was oven-dried at 60°C before weighing, and it was wetted before the experiment. Each hermit crab, after starving for 1 day, was cultured in an individual acrylic container (25 × 15 × 16 cm) with two trays of food, one containing 0.5 g (dry wt.) of seagrass debris and the other containing 0.5 g of dicot leaves. After 8 h, the food remaining was collected and dried before being weighed. The consumed weight was estimated by calculating the food weight difference before and after the experiment. The Mann–Whitney U test was used to compare the food consumed by the Dongsha and Siziwan crabs.

### Size increment comparison

To test whether food item differences between Dongsha and other sites contribute to size differences, the effect of seagrass debris and dicot leaves on growth rates was compared in hermit crabs collected from Dongsha and Siziwan. Forty male individuals (20 from Dongsha and 20 from Siziwan) were used. Ten from each site were fed seagrass debris and the remaining 10 were fed dicot leaves. A 3-cm layer of fine sand was provided with a glass of water to prevent the crabs from becoming dehydrated. The food provided to each individual was either 0.5 g (dry weight) of seagrass debris or dicot leaves, with water and food refilled every 3 days. Their shield length increments were calculated by the difference between March 18 and June 19, 2015 (the beginning and end of the experiment, respectively).

### Distribution

Because the baited traps attracted all species of land hermit crabs, potential competition among species could be evaluated by assessing the densities and niches of these sympatric species. Niche differences (i.e., distance from shore and elevation of habitats) were compared among species by using GPS coordinates and Google Earth. All the hermit crabs captured in the same traps were considered to live in the same neighborhood, and the location of the traps was assumed to be an indicator of their activity center. Whether different species occupied the same niche (traps) was determined using Chi-square tests.

For *C*. *rugosus*, the number of individuals caught in each trap was used as an index of local densities. Comparing island populations could reveal whether Dongsha has lower densities than elsewhere.

### Parasite

For this study, the hermit crabs on Dongsha and Siziwan were haphazardly collected at dusk within a week (June 30–July 2, 2014 on Dongsha and July 4–6, 2014 at Siziwan). Subsequently, the bodies and gills of the hermit crabs were individually examined for ectoparasites.

The collection of specimens was approved by Marine National Park Authority (Dongsha), Kenting National Park Authority (Howan), and the Agriculture Department of Penghu County (Tongpanyu, Huayu, Giang-jun-ou-yu and Yuanbeiyu), respectively; no permit was required for other locations, i.e., Siziwan, Xiaoliuqiu and Lanyu. The experiments on campus were approved by Institutional Animal Care and Use Committee (IACUC) of National Sun Yat-sen University.

## Results

### Size composition

**Fig 3:**
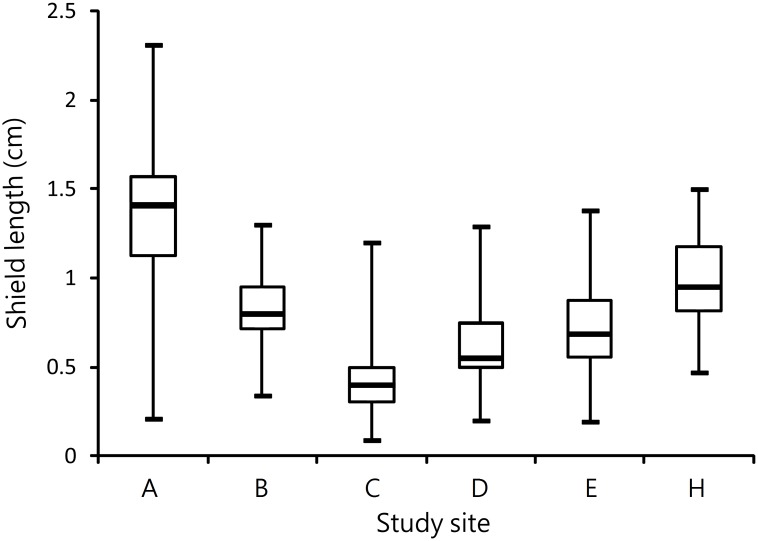
*Coenobita rugosus*. Comparison of body sizes (shield lengths) at different sites. Whiskers indicate the entire ranges, boxes indicate the 25th and 75th percentiles of the groups, and lines indicate the median values. A: Dongsha, B: Siziwan, C: Xiaoliuqiu, D: Lanyu, E: Howan, and H: Giang-jun-ou-yu.

**Fig 4:**
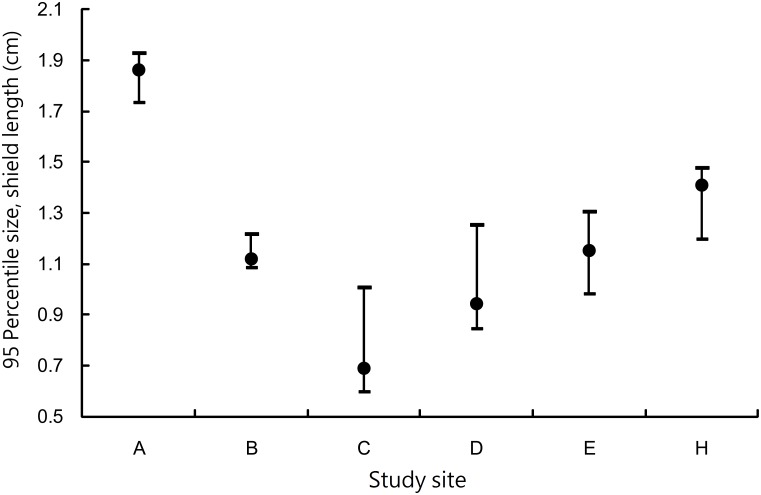
*Coenobita rugosus*. Comparison of the 95th percentile sizes of shield lengths from various islands. Error bars indicate 95% confidence intervals estimated using the resampling method. A: Dongsha, B: Siziwan, C: Xiaoliuqiu, D: Lanyu, E: Howan, and H: Giang-jun-ou-yu.

Nine islands were investigated for *C*. *rugosus*. Three islands had only one or two individuals, which was too few for further analysis. The other islands had 45–244 individuals. The largest individual, with a shield length of 2.31 cm, was collected on Dongsha. The maximum and median sizes were both greater on Dongsha than other islands (Fig 3). The 95th percentile size on Dongsha was 1.86 cm (95% CI.: 1.74–1.93 cm, n = 244), significantly greater than that of second-ranked Giang-jun-ou-yu (1.41 cm; 95% CI: 1.20–1.48 cm, n = 61) and those of Siziwan (1.12 cm; 95% CI: 1.09–1.22 cm, n = 62), Xiaoliuqiu (0.69 cm; 95% CI: 0.60–1.01 cm, n = 81), Lanyu (0.94 cm; 95% CI: 0.85–1.26 cm, n = 45), and Howan (1.15 cm; 95% CI: 0.99–1.31 cm, n = 98, Fig 4). Only a few individuals at the latter five islands exceeded the median size of those on Dongsha (Fig 3).

### Shell status

The conditions of the shells used by the crabs differed among the islands. The advantageous–disadvantageous ratio of 62:50 on Dongsha was the lowest, with Xiaoliuqiu, Lanyu, Howan, Siziwan, and Giang-jun-ou-yu exhibiting ratios of 62:19, 37:8, 70:28, 50:33, and 38:23, respectively; comparison with the Dongsha ratio revealed significant differences for all of the islands except Siziwan (P < 0.01, <0.01, = 0.02, = 0.59, and = 0.47, respectively, Chi-square tests; Table 1).

**Table 1:** *Coenobita rugosus*. Comparison of shell conditions at six sites. All comparisons are against Dongsha.

Site	Advantage	Disadvantage	% Adv.	P value (Chi-square)
Dongsha	62	50	55	
Xiaoliuqiu	62	19	77	<0.01
Lanyu	37	8	82	<0.01
Howan	70	28	71	0.02
Siziwan	50	33	60	0.59
Giang-jun-ou-yu	38	23	62	0.47

Because the increment rates of hermit crabs, estimated as the difference between the first and second cohorts, differed between islands (see later), further analysis of their shells was conducted, with the comparison restricted to individuals with sizes between the means of the first and second cohorts (i.e., 0.7–1.5 cm for Dongsha and 0.79–1.1 cm for Siziwan). The shell hypothesis predicts that the Dongsha population should have a more desirable shell status in these ranges. However, no dependence was found between shell status and site (35:27 on Dongsha and 22:15 at Siziwan; P = 0.93, Chi-square test) in these limited ranges, indicating that shell conditions do not differ in hermit crabs of intermediate size among the two islands.

### Cohort analysis

**Fig 5:**
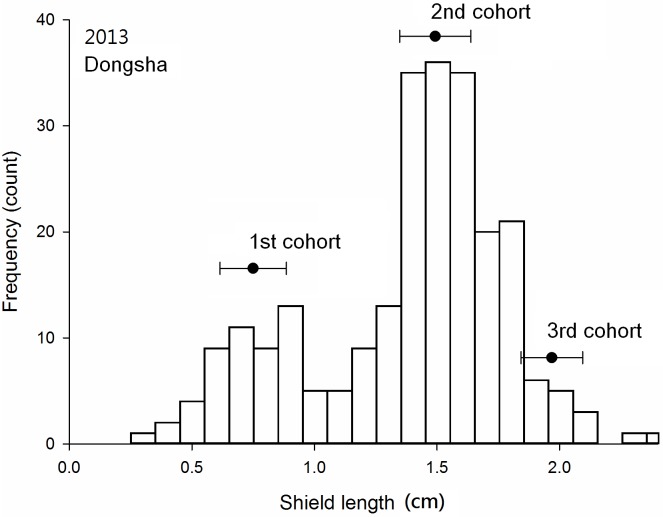
*Coenobita rugosus*. Size structures and cohort analyses for Dongsha in 2013. Black dots indicate the means and standard deviations of each cohort, as estimated using FISAT II.

**Fig 6:**
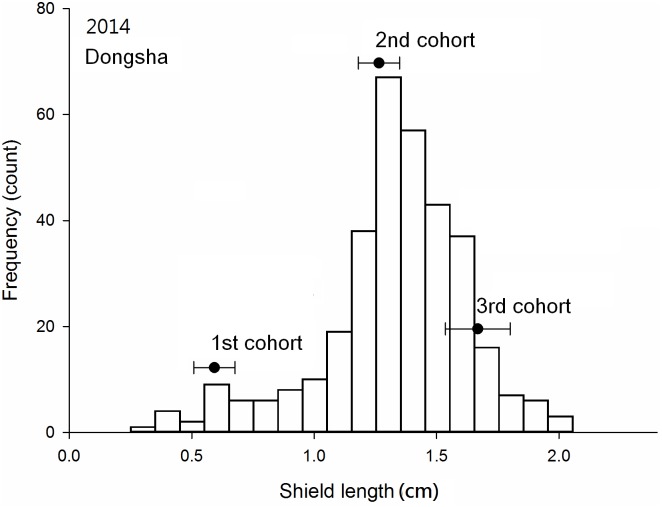
*Coenobita rugosus*. Size structures and cohort analyses for Dongsha in 2014. See Fig 5 for other legend.

**Fig 7:**
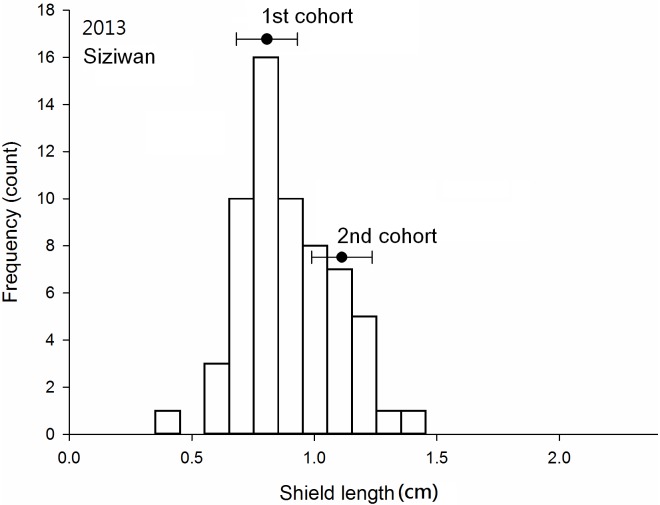
*Coenobita rugosus*. Size structures and cohort analyses at Siziwan in 2013. See Fig 5 for other legend.

**Fig 8:**
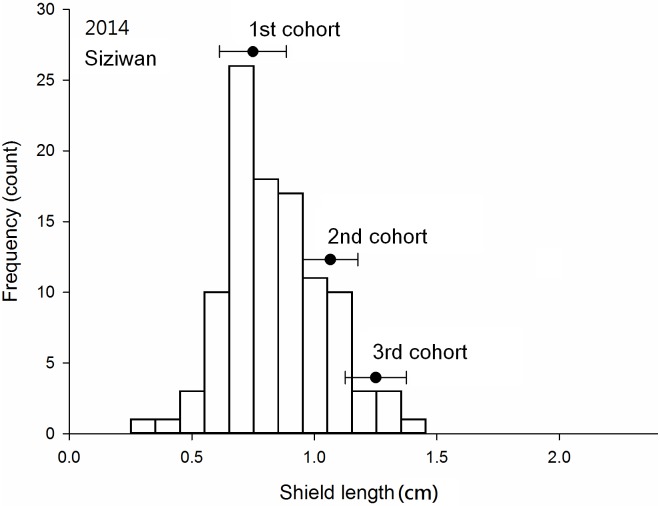
*Coenobita rugosus*. Size structures and cohort analyses at Siziwan in 2014. See Fig 5 for other legend.

Three cohorts were obvious in the Dongsha samples collected in November of both 2013 and 2014 (Figs 5 and 6); at Siziwan, two cohorts in November 2013 and three cohorts in November 2014 were identified (Figs 7 and 8). The cohort structures could be more clearly distinguished when examined using their own bins with respective intervals and ranges; however, for comparison purposes, all four used the same bins in the figures. The number of individuals in each cohort was estimated objectively by using FISAT II. In 2013, 8% of the Dongsha hermit crabs belonged to the third cohort, whereas none were in this year class at Siziwan. In 2014, the third cohort represented 16% (n = 314) and 4% (n = 99) for Dongsha and Siziwan, respectively (Table 2). The cohort structures were significantly dependent on site in both years, though this dependence was much stronger in the first cohort of Siziwan than of Dongsha (P < 0.01, Chi-square test). When analyses were performed within sites, the cohort structures were similar for Siziwan between 2013 and 2014 (P = 0.26, Chi-square test), but were dependent on year for Dongsha (P < 0.01, Chi-square test).

**Table 2:** *Coenobita rugosus*. Cohort structures at two sites according to FISAT II estimation of cohort composition.

2013 cohorts	Dongsha	%	Siziwan	%
1^st^	40	19	43	69
2^nd^	153	73	19	31
3^rd^	17	8	0	0
2014 cohorts				
1^st^	18	6	68	69
2^nd^	245	78	27	27
3^rd^	51	16	4	4

Chi-square test: Between sites, P < 0.01 in both years.

The means of the first cohorts were 0.70 cm (SD = 0.14 cm, 2013, Fig 5) and 0.63 cm (SD = 0.07 cm, 2014, Fig 6) on Dongsha and 0.80 cm (SD = 0.11 cm, 2013, Fig 7) and 0.73 cm (SD = 0.11 cm, 2014, Fig 8) at Siziwan. The means of the second cohorts were 1.52 cm (SD = 0.15 cm, 2013) and 1.34 cm (SD = 0.07 cm, 2014) on Dongsha and 1.09 cm (SD = 0.10 cm, 2013) and 1.05 cm (SD = 0.09 cm, 2014) at Siziwan. The means of the third cohorts were 1.96 cm (SD = 0.13 cm, 2013) and 1.66 cm (SD = 0.10 cm, 2014) on Dongsha and 1.22 cm (SD = 0.10 cm, 2014) at Siziwan.

**Fig 9:**
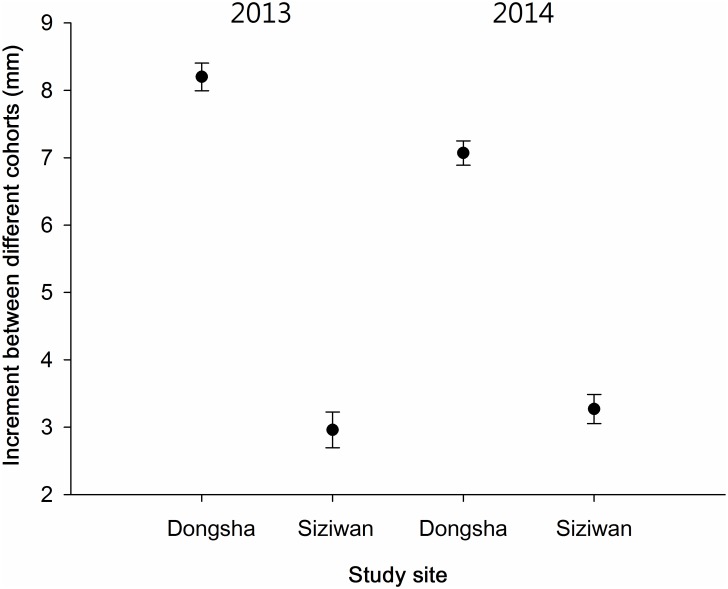
*Coenobita rugosus*. Comparison of size difference (in shield lengths) between cohorts 1 and 2.

**Fig 10:**
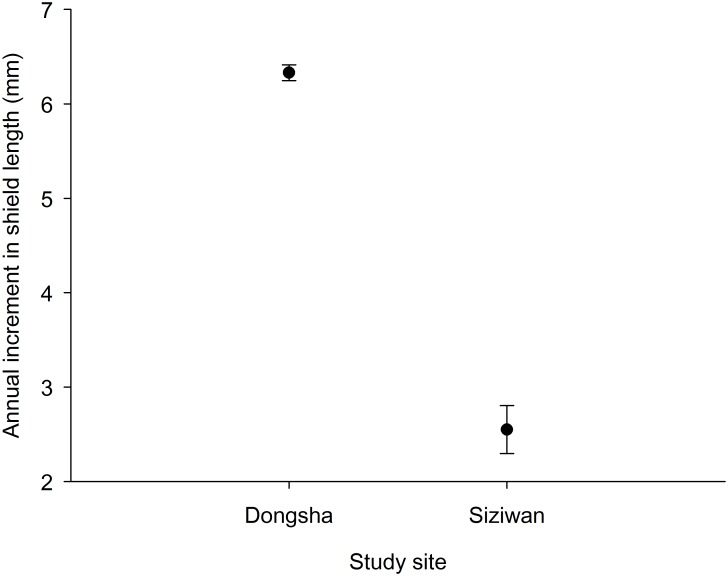
*Coenobita rugosus*. Comparison of annual increment of the first cohort of 2013 with the second cohort in 2014 for Dongsha and Siziwan.

The estimated size increments between the first and second cohorts using the first horizontal approach, within the year, were significantly greater for Dongsha (2013: 8.2 ± 0.2 (CI), 2014: 7.1 ± 0.2 mm) than for Siziwan (2013: 3.0 ± 0.3, 2014: 3.3 ± 0.2 mm) in both years (P < 0.01, *t* test, Fig 9). Similar results were obtained when using the second vertical approach, across years, to assess annual growth increment; the hermit crabs in the first cohort of 2013 increased by 6.33 ± 0.08 mm on Dongsha, whereas those at Siziwan increased by only 2.55 ± 0.25 mm—a significant difference (P < 0.01, *t* test, Fig 10).

### Condition indices

**Fig 11:**
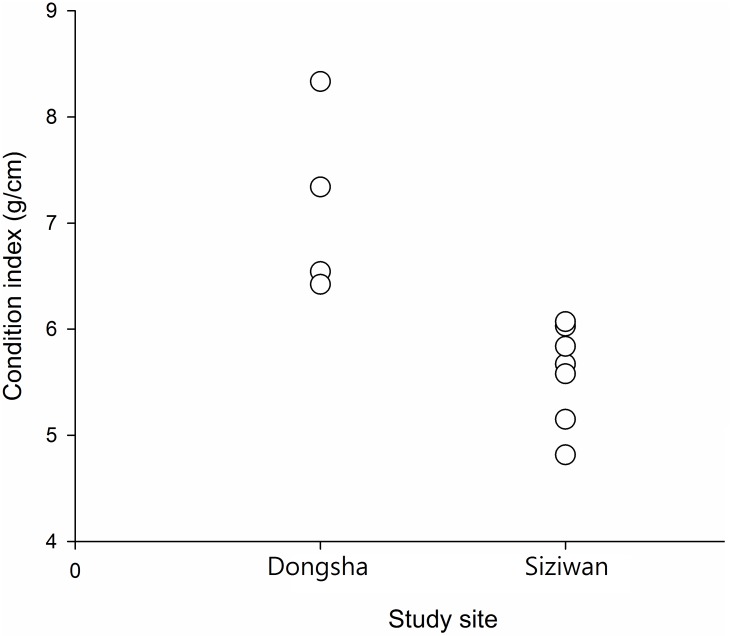
*Coenobita rugosus*. Comparison of the condition indices of males on Dongsha (n = 5) with those at Siziwan (n = 8). (P < 0.01, Mann–Whitney U test). Data points may overlap in the figure.

In a preliminary test, hermit crabs with a shield length of 10–11 mm both on Dongsha and at Siziwan were chosen for condition index comparison. All five individuals from Dongsha had condition indices of > 6, whereas six out of the eight individuals from Siziwan had condition indices of < 6, with the remaining two having condition indices of 6.02 and 6.07 (Fig 11). The difference between the two sites was significant (P < 0.01, Mann–Whiney U test).

**Fig 12:**
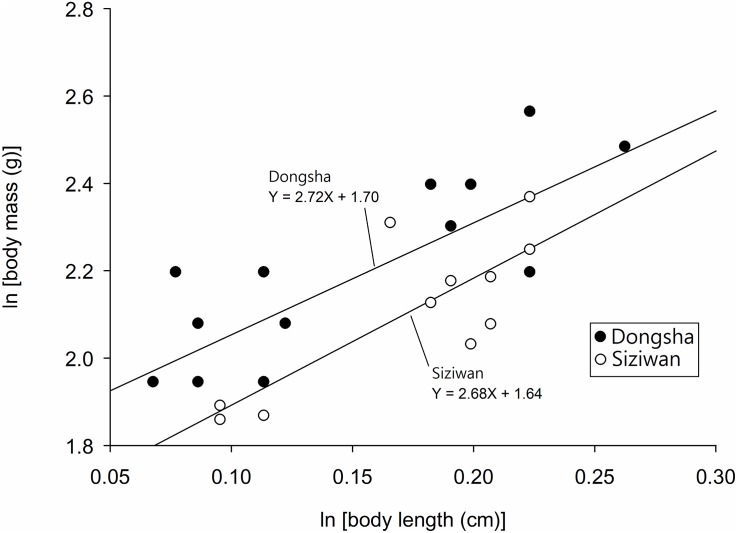
*Coenobita rugosus*. Comparison of the condition indices of Dongsha and Siziwan males. The slopes of the two populations are not significantly different (P = 0.69, ANCOVA); the intercepts, however, are significantly different (P = 0.03, ANCOVA), with the Dongsha samples exhibiting a greater intercept.

Because the condition index changes with size, we used ANCOVA to compare the log-transformed data of males from the two sites. Females were excluded from this analysis to avoid possible complications caused by gravid females. The slopes of transformed data did not differ significantly between the two sites (P = 0.69), but the intercepts of the two did (P = 0.03, ANCOVA, Fig 12), with the Dongsha population having a greater intercept (i.e., greater condition indices for same-sized hermit crabs in the Siziwan population).

### Food preference test

**Fig 13:**
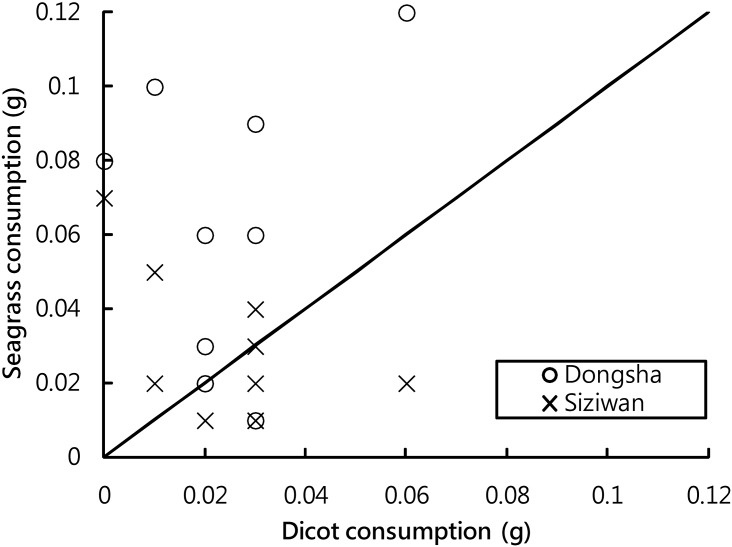
*Coenobita rugosus*. Results of food preference test of hermit crabs collected from Dongsha and Siziwan. A significant difference was found in the Dongsha crabs but not in the Siziwan crabs (P = 0.015 and P = 0.83, respectively, Wilcoxon signed-rank test). The diagonal line indicates a 1:1 dietary consumption.

During the 8-h experiment, up to 0.12 g dry weight of seagrass debris and up to 0.06 g of dicot leaves was consumed by each individual. The hermit crabs from Dongsha consumed significantly more seagrass debris than dicot leaves (P = 0.015, Wilcoxon signed-rank test); only one individual ate more dicot leaves during the experiment. No such difference in food preference was observed in the hermit crabs collected from Siziwan (P = 0.83, Wilcoxon signed-rank test, Fig 13).

### Size increment comparison

**Fig 14:**
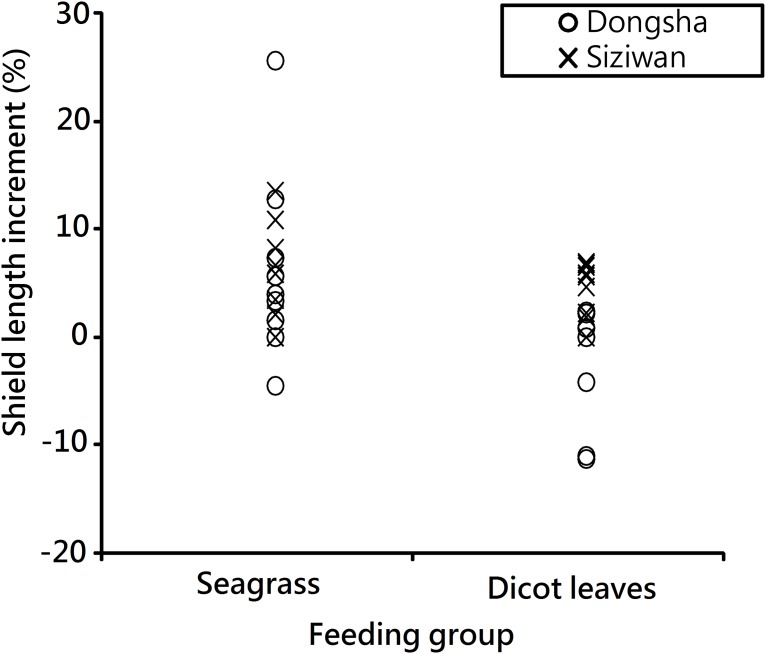
*Coenobita rugosus*. Comparison of size increment of crabs from Dongsha in the growth experiment. P < 0.01 for Dongsha crabs, P = 0.27 for Siziwan crabs, Mann-Whitney U Tests.

In an additional test lasting 3 months, most hermit crabs in the seagrass group (n = 8) from Dongsha had positive growth, with increments between −4.5% and 25.6%, but the dicot leaf group (n = 9) mostly experienced negative growth (ranging from −11.3% to 2.4%; P < 0.01, Mann–Whitney U test, Fig 14). All the hermit crabs from Siziwan had positive increments, and no significant difference in increments was observed between the food treatment groups (P = 0.27, Mann–Whitney U test).

For the same data set, but comparing crabs from the two sites that consumed the same food items, a significantly higher increment rate was found in the Siziwan crabs when fed dicot leaves (P<0.01, Mann–Whitney U test). No site difference in increment rates was found in the crabs fed seagrass. The statistical results are presented in Table 3.

**Table 3:** P values of Mann–Whitney U tests comparing crabs according to diet and location. Underlined categories indicate greater increments in cases of significant difference.

Food\Crab from	Dongsha		Siziwan
Seagrass		0.49	
	<0.01		0.27
Dicot		<0.01	

### Distribution

In interspecies assessment, hermit crabs from all sites were pooled. *C*. *rugosus* was found to be distributed closer to shore, and the other two hermit crab species (*C*. *cavipes* and *C*. *brevimanus*) were observed to be farther from shore. Of the 533 *C*. *rugosus* caught, 12% were > 35 m from shore; much higher percentages were recorded for *C*. *cavipes* (65%, n = 60) and *C*. *brevimanus* (69%, n = 29). The horizontal distribution pattern was found to be dependent on species (P < 0.01, Chi-square tests against *C*. *cavipes* and *C*. *brevimanus*, Table 4).

**Table 4:** Horizontal distribution of *Coenobita* species at the shore.

Species	Horizontal distance from the shore		P–value (χ2-test) against C. r.
	< 35 m	> 35 m	
*C*. *rugosus*	470	63	
*C*. *cavipes*	21	39	< 0.01
*C*. *brevimanus*	9	20	< 0.01

A similar analysis of habitat altitude was conducted to compare the three species. Proportionally more *C*. *rugosus* individuals were distributed at relatively lower elevations than were individuals of the other two. When 20 m was used as a cutoff altitude, 99% of *C*. *rugosus* were at a low elevation (n = 533), whereas only 45% of *C*. *cavipes* (n = 60) and 17% *C*. *brevimanus* (n = 29) were close to the sea level. A significant dependence was found between altitude distribution and species (Table 5, P < 0.01, Chi-square tests).

**Table 5:** Altitudinal segregation of *Coenobita* spp.

Species	Elevation from sea surface		P value (χ2-test) against *C*.*r*.
	< 20m	> 20m	
*C*. *rugosus*	527	6	
*C*. *cavipes*	27	33	< 0.01
*C*. *brevimanus*	24	5	< 0.01

Despite a certain extent of spatial segregation among the three *Coenobita* species, some overlap occurred among them. For example, some *C*. *rugosus* individuals were caught along with individuals of other *Coenobita* species. We used this as an index to test whether the *C*. *rugosus* individuals on Dongsha were less likely to encounter other hermit crab species. The index ranged between 0% and 67% among the six sites (Table 6); 14% of individuals were caught with other species on Dongsha, which was higher for all the other sites except Howan.

**Table 6:** *Coenobita rugosus*. Number of individuals with and without congeners in the same traps.

Site	With other species in the same traps	Without other species in the same trap	% with	P-value (χ2-test, against Dongsha)
Dongsha	35	209	14	
Giang-jun-ou-yu	0	61	0	<0.01
Xiaoliuqiu	1	80	1	<0.01
Lanyu	1	44	2	0.04
Siziwan	6	56	10	0.45
Howan	66	32	67	<0.01

**Fig 15:**
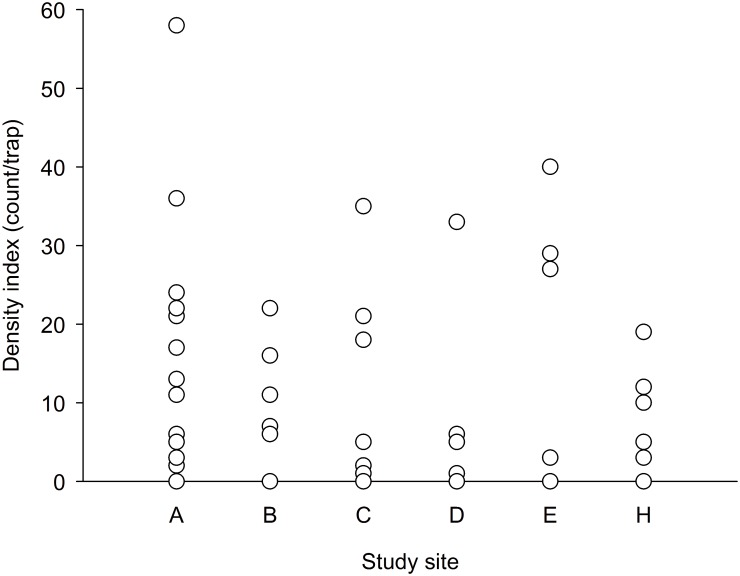
*Coenobita rugosus*. Comparison of density index (count/trap) at each study site (P = 0.65 with empty traps omitted or 0.22 when including all traps, Kruskal–Waillis tests). A: Dongsha, B: Siziwan, C: Xiaoliuqiu, D: Lanyu, E: Howan, and H: Giang-jun-ou-yu.

We used the numbers of hermit crabs caught in each trap as an index of local densities, and we compared the results of various islands in the hope of determining whether Dongsha had generally lower densities and thus lower intraspecies competition. The local densities of *C*. *rugosus* within a site were highly variable among traps (Fig 15). The highest density (i.e., 58 individuals in a trap) was recorded on Dongsha. A nonparametric comparison did not reveal significant differences in the local densities of the six study sites (P = 0.65 with empty traps omitted or 0.22 when all traps were included, Kruskal–Waillis tests).

### Parasite

Between June 30 and July 6, 2015, a total of 56 male and 48 female hermit crabs from Dongsha and 43 male and 22 female hermit crabs from Siziwan were collected for ectoparasite examination. No parasite was found on the Dongsha crabs, but three hermit crabs, two males and one female, from Siziwan were infected by unidentified ectoparasites. These infected individuals were not smaller than those uninfected hermit crabs examined in the condition index analysis for the same site (unpublished, data available in Hsu 2015).

## Discussion

The population of the land hermit crab *C*. *rugosus* reached larger sizes on Dongsha than on other islands around Southern Taiwan. The evidence obtained in this investigation clearly falsified some hypotheses.

The first hypothesis favored before the testing was that the availability of shells determines the growth of hermit crabs at an island, because gastropod shells could constrain the growth of hermit crabs [21, 29, 30, 34, 35]. Thus, Dongsha, which was found to have the largest *C*. *rugosus* individuals, is predicted according to the shell hypothesis to have the most favorable shells among all the islands investigated. A direct survey of the shells available to hermit crabs is straightforward but difficult [36]; moreover, the shells accessible to human investigators are not necessarily the same as those available to the hermit crabs, and vice versa.

The method we chose was to examine the actual shells used by extant hermit crabs. Presumably, more individuals would use adequate shells on islands where gastropod shell supply is supposed to be plentiful.

The result that the shell status on Dongsha was not more favorable than that on the other islands was surprising. We considered whether the index used was inadequate. However, such an explanation would seem likely only in the event of nonsignificant differences; by contrast, we found significantly poorer shell status on Dongsha in three out of five comparisons (and insignificant difference in the other two comparisons), contradicting the prediction of the shell hypothesis.

The *C*. *rugosus* populations on islands other than Dongsha with more favorable shell conditions were not constrained by the shells they carried, with most having enough space to grow further within their shells. By contrast, on Dongsha, the hermit crabs outgrew their shells. Therefore, although shell adequacy at individual level could influence hermit crab growth, it is not an appropriate explanation of large body sizes at the population level. The more favorable shell conditions at sites of small hermit crabs, or poor shell conditions at Dongsha, where body sizes were large, is strong evidence refuting the adequacy of shell-constraint hypothesis.

Do other islands also have abundant large but less protective shells? The large shells carried by Dongsha *C*. *rugosus* were mostly of African giant snails (*Achatina fulica*). This is a widely distributed species in Taiwan (personal observation). Their shells are typically thinner and more brittle than those of marine gastropods. Hermit crabs on islands other than Dongsha may simply not be large enough to require shells of *A*. *fulica*, because most of them had adequate shells to suit their sizes.

The logical deduction from these results is that the relatively poor shell status on Dongsha is not the cause, but could be the result of large hermit crab size. The shell-constraint hypothesis does predict that if Dongsha had larger, more favorable shells, the *C*. *rugosus* on the island could reach even larger sizes. To properly explain the large size of *C*. *rugosus* on this island, other mechanisms should be examined.

The hypothesis of parasitism was not supported in this study because only three of the 169 investigated individuals carried ectoparasites. Furthermore, these three individuals did not exhibit poor condition indices for their sizes [28]. Despite this, we cannot rule out the possible roles of endoparasites, which we did not examine. However, there is no reason to suspect that hermit crabs on Dongsha would have a lower prevalence of endoparasites than those on other islands.

The competition that *C*. *rugosus* may encounter has two sources: interspecific and intraspecific [37]. As revealed by our spatial analyses, segregation exists among congeneric hermit crab species. The distribution of C. *rugosus* is closer to shore lines than are those of other species on the same islands (Tables 4 and 5). This segregation in space should reduce potential niche overlap among hermit crab species. Dongsha ranked high among the study isalnds in our analysis estimating the frequencies of *C*. *rugosus* on the basis of the number caught in traps with congeners (Table 6). Therefore, *C*. *rugosus* are more likely to encounter interspecific competition on Dongsha than on most other islands of this study—a finding incompatible with the predictions of the interspecific competition hypothesis.

Possible intraspecific competition was assessed according to the numbers of hermit crabs caught in individual traps. However, the observed density variation among islands was not significantly higher than that within islands (Fig 15). The highly mobile *C*. *rugosus* are obviously opportunistic in food searching, because one baited trap on Dongsha attracted as many as 58 hermit crabs. Whatever food items may be available on the shore, they are potentially accessible to many individuals moving on the shore [38]. Although no fighting for food was observed, interference competition is possible because fighting for shells was seen (e.g., on Dongsha). The degree of intraspecific competition as experienced by individuals must be highly variable within islands as implied from numbers of neighbors one has (Fig 15). The present conclusion is that no evidence was found suggesting less competition on Dongsha than on the other islands.

Two hypotheses (i.e., predation and food availability) remain unevaluated. The cohort structures revealed three year classes on Dongsha, but at Siziwan, where *C*. *rugosus* had relatively small sizes, only minor third cohorts or none at all were found. The larger body sizes on Dongsha could be due to longer life spans, which in turn may be a result of a lack of predation and/or better food availability.

Actual surveys and comparisons of predators of hermit crabs are difficult. Monkeys, birds, and land crabs are listed as potential predators of hermit crabs [39]. Unlike for our other hypotheses, no experiments or observations were designed to specifically test the predator hypothesis in this investigation. We raise two related points here. First, humans may be a source of hermit crab mortality. Because of its relatively small size, *C*. *rugosus* is not a favored species in the pet market (personal observation of CHH). Second, rats and birds are considered potential predators of land hermit crabs. We noticed many rats in garbage dumping areas on Dongsha, but no quantitative comparison of the islands was conducted. One could suggest that mortality rates of *C*. *rugosus* were lower on Dongsha than on the other study islands. But there is no reason to suspect that there were fewer predators on Dongsha.

Three-year-old hermit crabs represented only a small proportion of the individuals investigated in the study. Clearly, a greater proportion of the population could reach 3 years old on Dongsha than at Siziwan (Table 2). Although this phenomenon could be a result of a lack of predation, the significantly larger sizes of the 2-year-old cohorts on Dongsha than at Siziwan is not a prediction of the predation hypothesis. Hence, some other reasons, e.g., food conditions, may be involved.

How *C*. *rugosus* grows faster on certain islands, as predicted by the food hypothesis, requires further consideration. Because Dongsha had abundant seagrass debris accumulated on the shore, whereas the other islands did not, one reasonable deduction is that the seagrass debris is a high-quality food for hermit crabs.

Among marine plants, seagrass may not be a preferred food source for many herbivores and thus most may be consumed in detrital forms in the sea [40, 41]. Once washed ashore, partially decomposed seagrasses may be a more desirable food source, e.g., especially when compared to dicot leaves also available on the beaches.

Two experiments designed in this study further tested the food hypothesis as a possible explanation for the large size of *C*. *rugosus* on Dongsha. The first experiment tested whether the hermit crabs preferred seagrass debris when given a choice between it and dicot leaves. The dicot leaves were found predominant on all the other islands but represented, at most, a small proportion on Dongsha (personal observation of the authors). Our experiment indicated that only the Dongsha hermit crabs preferred seagrass, with no such preference observed in the Siziwan crabs. Although this result for Dongsha is compatible with the prediction of the food hypothesis, the nonsignificant results at Siziwan suggest that factors other than food quality might also influence how much the hermit crabs ate in our preference experiment.

The growth experiment comparing hermit crabs fed different food also had positive and significant results for the hermit crabs collected from Dongsha but not for those from Siziwan. Specifically, the Dongsha hermit crabs grew faster when fed seagrass debris they preferred; however, the Siziwan hermit crabs did not exhibit any response in preference or growth rate when fed different food items. We suggest that acclimation to poor-quality food (i.e., dicot leaves) might occur at Siziwan, where crabs were used to eating dicot leaves, the only food available. They acclimated to the food and digested dicot leaves efficiently. This might be through acclimation of their own enzyme systems or through the change in microbial communities in their digestive systems. One piece of supporting evidence for this acclimation hypothesis is that the increment rate is higher at Siziwan than on Dongsha for the two groups both feeding on dicot leaves (Table 3). Those crabs from Dongsha usually had negative growth increments when fed dicot leaves during the experimental period, whereas those from Siziwan all exhibited positive increments. Clearly, when no other food sources are available, *C*. *rugosus* may require some time to adjust to dicot leaves. The duration of acclimation may be a crucial factor. Our growth experiment lasted three months, and the diet effect was significant between Dongsha and Siziwan crabs. Obviously, an acclimation period longer than 3 months is needed. Once acclimated to the poor-quality food, the hermit crab could grow, even at comparable rates to those fed seagrasses; this is demonstrated in the comparison between food treatments of Siziwan crabs (Fig 14). The long acclimation duration may thus contribute to their small body sizes in islands without seagrass debris. During this acclimation period, the crabs presumably lag behind in body sizes. Additional analyses comparing the composition of the two food sources available on the shores could potentially reveal what the critical ingredient causing the differences in growth rates is.

The food hypothesis alone could explain three traits of *C*. *rugosus* on Dongsha, namely faster growth, larger sizes, and longer lives. The predation hypothesis may have played a role, but could not explain the food preferences and faster growth rates of the *C*. *rugosus* individuals on Dongsha.

We conclude that food quality, particularly the availability of piles of seagrass debris on the shore, is the most likely factor causing *C*. *rugosus* to grow larger on Dongsha than on other islands around Southern Taiwan. This represents a situation in which the productivity of marine habitats affects populations on land.

That the seagrass beds not only contribute to marine productivity (e.g., commercial fisheries of penaeid shrimp [42–44]) but also to terrestrial populations (e.g., the land hermit crabs on the shore in this study) is unexpected. Clearly, more remains to be studied about the contribution of seagrass, e.g., after their leaves were shed and transported elsewhere [41]. The contribution of islands with seagrass beds may be more crucial than those islands without them because the primary production of seagrass beds may be consumed elsewhere. These seagrass beds are threatened by local and global changes [45, 46], the negative effects of which could also extend beyond marine ecosystems to at least the land hermit crabs living near the shore.

## Supporting information

S1 Supporting Information Data used in figures are included in supporting information for potential alternative analyses. (DOCX)
