# Supplementary material for: Mechanisms causing size differences of the land hermit crab Coenobita rugosus among eco-islands in Southern Taiwan
Source: PLoS One. 2017 Apr 7;12(4):e0174319. doi: 10.1371/journal.pone.0174319 (PMC5384810; doi:10.1371/journal.pone.0174319)
Supplement: S1 Supporting Information — (DOCX) [file pone.0174319.s001.docx]

**Data corresponding to Fig 1. The measure of *Coenobita rugosus* of shield length in maximum, mean, minimum for each sites.**

| **Site** | Max (cm) | Mean (cm) | Min (cm) | Count (n) |
| --- | --- | --- | --- | --- |
| A: Dongsha | 2.31 | 1.31 | 0.21 | 244 |
| B: Siziwan | 1.30 | 0.87 | 0.34 | 62 |
| C: Hsioliuchiu | 1.20 | 0.64 | 0.09 | 81 |
| D: Lanyu | 1.29 | 0.71 | 0.20 | 45 |
| E: Howan | 1.38 | 0.71 | 0.19 | 98 |
| F: Tongpanyu | 0.96 | 0.955 | 0.95 | 2 |
| G: Huayu | 0.95 | 0.95 | 0.95 | 1 |
| H: Giang-jun-ou-yu | 1.50 | 1.00 | 0.47 | 61 |
| I: Yuanbeiyu | 1.07 | 1.07 | 1.07 | 1 |

**Data corresponding to Fig 2. *Coenobita rugosus*. Comparison of the 95 percentile sizes of shield lengths from various islands.** 95% confidence intervals estimated by resampling method.

| **Site** | Upper (cm) | Middle (cm) | lower(cm) |
| --- | --- | --- | --- |
| A: Dongsha | 1.93 | 1.86 | 1.74 |
| B: Siziwan | 1.22 | 1.12 | 1.09 |
| C: Hsioliuchiu | 1.01 | 0.69 | 0.6 |
| D: Lanyu | 1.26 | 0.94 | 0.85 |
| E: Howan | 1.31 | 1.15 | 0.99 |
| H: Giang-jun-ou-yu | 1.48 | 1.41 | 1.2 |

**Data corresponding to Fig 3. *Coenobita rugosus*. The size structures and cohort analyses at Dongsha in 2013.** The means, standard deviations and counts were estimated by FISAT II.

| 2013  Dongsha | Mean (cm) | Standard deviation (cm) | Count (n) |
| --- | --- | --- | --- |
| 1st cohort | 0.7 | 0.14 | 40.4 |
| 2nd cohort | 1.52 | 0.15 | 152.99 |
| 3rd cohort | 1.97 | 0.13 | 16.78 |

**Data corresponding to Fig 4. *Coenobita rugosus*. The size structures and cohort analyses at Dongsha in 2014.** The means, standard deviations and counts were estimated by FISAT II.

| 2014  Dongsha | Mean (cm) | Standard deviation (cm) | Count (n) |
| --- | --- | --- | --- |
| 1st cohort | 0.63 | 0.07 | 18.07 |
| 2nd cohort | 1.34 | 0.07 | 244.87 |
| 3rd cohort | 1.66 | 0.11 | 50.82 |

**Data corresponding to Fig 5. *Coenobita rugosus*. The size structures and cohort analyses at Siziwan in 2013.** The means, standard deviations and counts were estimated by FISAT II.

| 2013  Siziwan | Mean (cm) | Standard deviation (cm) | Count (n) |
| --- | --- | --- | --- |
| 1st cohort | 0.8 | 0.11 | 43.19 |
| 2nd cohort | 1.09 | 0.1 | 19.27 |

**Data corresponding to Fig 6. *Coenobita rugosus*. The size structures and cohort analyses at Siziwan in 2014.** The means, standard deviations and counts were estimated by FISAT II.

| 2014  Siziwan | Mean (cm) | Standard deviation (cm) | Count (n) |
| --- | --- | --- | --- |
| 1st cohort | 0.73 | 0.11 | 67.59 |
| 2nd cohort | 1.05 | 0.09 | 26.52 |
| 3rd cohort | 1.23 | 0.1 | 3.88 |

**Data corresponding to Fig 7. *Coenobita rugosus.* Comparison of size difference (in shield lengths) between cohort 2 and 1.**

| **Year / Site** | | Increment between different cohorts (mm) | |
| --- | --- | --- | --- |
|  |  | Mean (mm) | SD (mm) |
| 2013 | Dongsha | 8.2 | 1.45 |
|  | Siziwan | 2.96 | 1.06 |
| 2014 | Dongsha | 7.07 | 1.49 |
|  | Siziwan | 3.27 | 1.07 |

**Data corresponding to Fig 8. *Coenobita rugosus*. Comparison of annual increment of the 1^st^ cohort of 2013 to 2^nd^ cohort in 2014, between Dongsha and Siziwan.**

| **Site** | Annual increment in shield length (mm) | |
| --- | --- | --- |
|  | Mean (mm) | SD (mm) |
| Dongsha | 6.33 | 0.72 |
| Siziwan | 2.55 | 1.07 |

**Data corresponding to Fig 9. *Coenobita rugosus.* Comparison of the Condition Index of males between Dongsha and Siziwan.**

| **Site** | Condition index (g/cm) | | |
| --- | --- | --- | --- |
|  | Mean (mm) | SD (mm) | Count (n) |
| Dongsha | 7.01 | 0.83 | 5 |
| Siziwan | 5.59 | 0.46 | 8 |

**Data corresponding to Fig 10. *Coenobita rugosus.* Comparison of the condition index of males between Dongsha and Siziwan.** Using ANCOVA.

| **Dongsha** | | **Siziwan** | |
| --- | --- | --- | --- |
| ln [body length (cm)] | ln [body mass (g)] | ln [body length (cm)] | ln [body mass (g)] |
| 0.09 | 2.08 | -0.05 | 1.5 |
| 0.37 | 2.71 | -0.45 | 0.47 |
| 0.35 | 2.56 | 0.1 | 1.89 |
| 0.22 | 2.56 | 0.07 | 1.71 |
| 0.33 | 2.48 | 0.34 | 2.58 |
| 0.44 | 2.83 | 0.22 | 2.37 |
| 0.58 | 3.14 | 0.32 | 2.42 |
| 0.07 | 1.95 | -0.05 | 1.68 |
| 0.4 | 2.77 | 0.2 | 2.03 |
| 0.2 | 2.4 | 0.21 | 2.19 |
| 0.26 | 2.48 | 0.18 | 2.13 |
| 0.28 | 2.2 | 0.01 | 1.81 |
| -0.16 | 1.39 | 0.29 | 2.39 |
| -0.29 | 0.69 | -0.62 | 0 |
| 0.11 | 1.95 | -0.29 | 0.9 |
| 0.09 | 1.95 | 0.34 | 2.44 |
| -0.2 | 1.1 | 0 | 1.74 |
| 0.22 | 2.2 | -0.02 | 1.47 |
| 0.67 | 3.4 | -0.05 | 1.48 |
| 0.46 | 3.14 | 0.29 | 2.29 |
| 0.48 | 3.14 | -0.21 | 1.39 |
| 0.34 | 2.64 | 0.24 | 2.31 |
| 0.42 | 3.04 | 0.17 | 2.31 |
| 0.12 | 2.08 | 0.22 | 2.25 |
| 0.57 | 3.3 | 0.1 | 1.86 |
| 0.33 | 2.56 | 0.3 | 2.54 |
| 0.27 | 2.48 | 0.31 | 2.47 |
| -0.2 | 1.1 | 0.21 | 2.08 |
| 0.56 | 3.22 | 0.25 | 2.43 |
| 0.4 | 2.71 | 0.31 | 2.53 |
| 0.37 | 2.71 | 0.11 | 1.87 |
| 0.11 | 2.2 | 0.27 | 2.38 |
| 0.29 | 2.4 | -0.17 | 1.08 |
| 0.18 | 2.4 | -0.05 | 1.41 |
| 0.08 | 2.2 | -0.53 | 0.07 |
| 0.42 | 2.89 | 0.02 | 1.74 |
| 0.36 | 2.71 | 0.35 | 2.62 |
| 0.6 | 3.26 | -0.21 | 1.07 |
| 0.34 | 2.64 | 0.03 | 1.6 |
| 0.56 | 3.04 | 0.2 | 2.3 |
| 0.44 | 2.94 | 0.19 | 2.18 |
| 0.64 | 3.3 | 0.06 | 1.78 |
| 0.46 | 3.09 |  |  |
| -0.29 | 0.69 |  |  |
| 0.11 | 1.95 |  |  |
| 0.09 | 1.95 |  |  |
| -0.2 | 1.1 |  |  |
| 0.22 | 2.2 |  |  |
| -0.29 | 0.69 |  |  |
| 0.39 | 2.56 |  |  |
| 0.34 | 2.56 |  |  |
| -0.19 | 1.39 |  |  |
| 0.41 | 2.94 |  |  |
| 0.19 | 2.3 |  |  |
| 0.54 | 3.18 |  |  |
| 0.59 | 3.14 |  |  |

**Data corresponding to Fig 11. *Coenobita rugosus.* Comparison of the preference between dicot leaves and seagrass.**

| **Dongsha** | | **Siziwan** | |
| --- | --- | --- | --- |
| Seagrass consumption (g) | Dicot  consumption (g) | Seagrass consumption (g) | Dicot  consumption (g) |
| 0.06 | 0.02 | 0.03 | 0.03 |
| 0.06 | 0.02 | 0.02 | 0.03 |
| 0.12 | 0.06 | 0.02 | 0.06 |
| 0.1 | 0.01 | 0.01 | 0.02 |
| 0.09 | 0.03 | 0.03 | 0.03 |
| 0.03 | 0.02 | 0.07 | 0 |
| 0.08 | 0 | 0.04 | 0.03 |
| 0.02 | 0.02 | 0.05 | 0.01 |
| 0.06 | 0.03 | 0.02 | 0.01 |
| 0.01 | 0.03 | 0.01 | 0.03 |

**Data corresponding to Fig 12. *Coenobita rugosus.* Comparison of size increment of crabs from Dongsha in the growth experiment.**

|  | Shield length increment (%) | | | |
| --- | --- | --- | --- | --- |
|  | Dongsha | | Siziwan | |
| **Treatment**  **/ Number of crab** | Seagrass | Dicot leaves | Seagrass | Dicot leaves |
| 1 | 3.36 | -4.17 | 8.25 | 6.93 |
| 2 | 5.66 | -11.29 | 3.48 | 1.87 |
| 3 | -4.50 | 0.00 | 13.54 | 2.25 |
| 4 | 7.37 | 2.20 | 6.67 | dead |
| 5 | dead | 0.00 | 10.87 | 5.88 |
| 6 | 1.56 | 2.38 | dead | 4.65 |
| 7 | 25.64 | .85 | dead | 6.56 |
| 8 | 12.79 | -11.01 | 5.88 | 5.66 |
| 9 | 3.97 | 0.00 | dead | dead |
| 10 | dead | Dead | 2.11 | 6.78 |
